# Severe COVID-19 is characterised by inflammation and immature myeloid cells early in disease progression

**DOI:** 10.1016/j.heliyon.2022.e09230

**Published:** 2022-04-01

**Authors:** Liam Townsend, Adam H. Dyer, Aifric Naughton, Sultan Imangaliyev, Jean Dunne, Rachel Kiersey, Dean Holden, Aoife Mooney, Deirdre Leavy, Katie Ridge, Jamie Sugrue, Mubarak Aldoseri, Jo Hannah Kelliher, Martina Hennessy, Declan Byrne, Paul Browne, Christopher L. Bacon, Catriona Doyle, Ruth O’Riordan, Anne-Marie McLaughlin, Ciaran Bannan, Ignacio Martin-Loeches, Arthur White, Rachel M. McLoughlin, Colm Bergin, Nollaig M. Bourke, Cliona O’Farrelly, Niall Conlon, Clíona Ní Cheallaigh, STAR COVID Bioresource

**Affiliations:** a Department of Infectious Diseases, St James's Hospital, Dublin, Ireland; b Department of Clinical Medicine, Trinity Translational Medicine Institute, Trinity College Dublin, Ireland; c Department of Medical Gerontology, Trinity Translational Medicine Institute, Trinity College Dublin, Ireland; d Department of Immunology, St James's Hospital, Dublin, Ireland; e Host Pathogen Interactions Group, School of Biochemistry and Immunology, Trinity College Dublin, Ireland; f School of Biochemistry and Immunology, Trinity College Dublin, Ireland; g Department of Intensive Care Medicine, St James's Hospital, Dublin, Ireland; h Clinical Research Facility, St James's Hospital, Dublin, Ireland; i Department of General Medicine, St James's Hospital, Dublin, Ireland; j Department of Haematology, St James's Hospital, Dublin, Ireland; k Department of Respiratory Medicine, St James's Hospital, Dublin; l School of Computer Science and Statistics, Trinity College Dublin, Ireland; m School of Medicine, Trinity College Dublin, Ireland; n Department of Immunology, School of Medicine, Trinity College Dublin, Dublin, Ireland

**Keywords:** COVID-19, Immune phenotype, Neutrophil maturity, Machine learning, Biomarkers

## Abstract

SARS-CoV-2 infection causes a wide spectrum of disease severity. Identifying the immunological characteristics of severe disease and the risk factors for their development are important in the management of COVID-19. This study aimed to identify and rank clinical and immunological features associated with progression to severe COVID-19 in order to investigate an immunological signature of severe disease. One hundred and eight patients with positive SARS-CoV-2 PCR were recruited. Routine clinical and laboratory markers were measured, as well as myeloid and lymphoid whole-blood immunophenotyping and measurement of the pro-inflammatory cytokines IL-6 and soluble CD25. All analysis was carried out in a routine hospital diagnostic laboratory. Univariate analysis demonstrated that severe disease was most strongly associated with elevated CRP and IL-6, loss of DLA-DR expression on monocytes and CD10 expression on neutrophils. Unbiased machine learning demonstrated that these four features were strongly associated with severe disease, with an average prediction score for severe disease of 0.925. These results demonstrate that these four markers could be used to identify patients developing severe COVID-19 and allow timely delivery of therapeutics.

## Highlights

• Severe COVID-19 is characterised by a combination of emergency myelopoiesis and inflammation.
• These changes can be rapidly identified in a diagnostic laboratory, facilitating intervention.
• This disease signature was derived from a cohort of patients with a wide range of ages, frailty and COVID-19 severity.

## Graphical abstract

**Figure:**
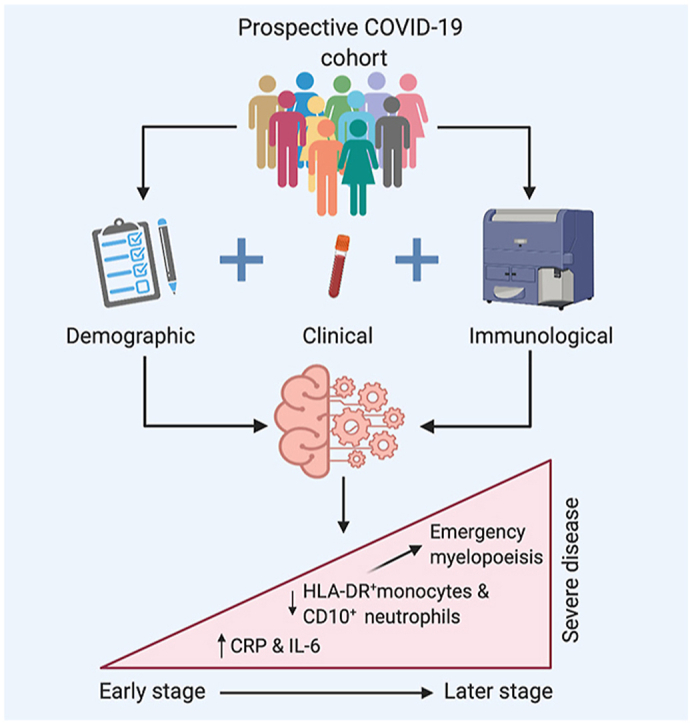

## 1 Introduction

COVID-19, caused by infection with the SARS-CoV-2 virus, is responsible for the current global pandemic [1]. The significant morbidity and mortality associated with this infection has placed unprecedented pressures on healthcare systems worldwide [2]. Numerous risk factors for severe disease have been identified in repeated meta-analyses. These include; older age, male sex, obesity and the presence of comorbidities such as chronic cardiac and respiratory diseases, cancer and primary and secondary immunodeficiency states [3, 4]. Even in the presence of several risk factors, the clinical course of SARS-CoV-2 infection is remarkably variable.

COVID-19 appears to have a biphasic pattern of illness [5, 6, 7]. In the early phase of infection, viral replication promotes an initial immune response. This is characterised by an elevation in pro-inflammatory cytokines and an influx of monocytes and T lymphocytes into the lungs [8, 9, 10, 11, 12]. A subsequent exuberant inflammatory phase develops in approximately 20% of individuals 6–10 days post symptom onset [13]. This phase is characterised by a deterioration in clinical parameters with increasing lung infiltrates and a rise in oxygen requirements [14, 15]. In approximately 5% of cases, progressive dyspnoea occurs, resulting in acute respiratory distress syndrome (ARDS) like picture and a requirement for mechanical ventilation [16]. Interestingly, the pathological changes are not limited to the respiratory system. Extra-pulmonary manifestations of COVID-19 are common and include gastrointestinal symptoms, elevated liver enzymes, and altered coagulation parameters and thrombotic events [17, 18, 19].

An important consideration in the evaluation of the pathophysiology of COVID-19 is identifying signatures that are predictive of severe disease, as well as identifying patient characteristics that are associated with more severe disease phenotypes. Careful assessment of temporally linked clinical and laboratory parameters is required to identify such features. Utilising multivariate analytic and machine learning approaches offers an exciting way of integrating large and complex data sets to identify important predictive features [26, 27, 28].

Recent reports have highlighted changes in myeloid and lymphoid compartments that can be associated with severe disease [[20], [22], [24], 25]. The relative importance of these findings in relation to other important risk factors that can influence trajectories of disease progression is underexplored. Finally, the rapid and early identification of those at risk of severe disease is essential for the prompt administration of immunotherapies. Both dexamethasone and tocilizumab have been shown to reduce mortality in patients requiring oxygen therapy [29, 30]. However, administering this in a timely fashion has proven difficult [31].

Identification of patients at risk of severe disease and in need targeted treatment is complex and requires the availability of prompt objective laboratory indices. In order to be effective parameters identified in research studies need to be readily measurable in diagnostic laboratories so that results can be applied in prompt clinical decision making.

This study aimed to assess the clinical and immunological features of COVID-19 disease and identify the characteristics of severe disease. We collected demographic data, immunophenotyping of myeloid and lymphoid populations, measurement of pro-inflammatory cytokines, and routine clinical laboratory parameters of patients with confirmed polymerase-chain reaction (PCR) positive SARS-CoV-2 infection. A machine learning model was applied to this dataset to identify a predictive disease signature of severe COVID-19.

## 2 Results

### 2.1 Participant characteristics

One-hundred and eight participants were recruited (42/108 (38.9%) female). Baseline characteristics are shown in Table 1. Seventeen had mild disease, 52 had moderate disease (of whom 30 required supplemental oxygen) and 39 had severe disease (Table 2). Of those with severe disease, 26/39 were admitted to ICU for intensive monitoring and mechanical ventilation and an additional 13/39 patients were assessed for ICU admission for mechanical ventilation but were not admitted to ICU due to high likelihood of non-survival. Ten patients with severe COVID-19 died (2/26 admitted to ICU and 8/13 assessed but not admitted to ICU). The median inpatient stay for those with moderate/severe disease was 11.5 days (range 2–108). The median length of stay in ICU was 12 days (range 1–39). Disease characteristics are shown in Table 2, while baseline laboratory parameters are shown in Table 3. As shown in Supplemental table 2, clinical blood tests were conducted at a median of six days post symptom onset and, for those requiring supplemental oxygen, a median of 1.5 days prior to peak oxygen requirement, which was deemed to be a marker of peak respiratory illness.

**Table 1:** Baseline demographics.

Characteristic	
Total number of patients	108
Admitted to hospital, number of patients (percentage of total)	91 (84%)
Mild disease	17 (16%)
Moderate disease	52 (48%)
Severe disease	39 (36%)
Date of first SARS-CoV-2 positive sample, range	10/3/2020-2/5/2020
Interval between symptom onset and peak oxygen requirement, median (range), days	8 (2–15)
Requirement for supplemental oxygen, number of patients (% of total)	65 (60%)
Peak CRP, median (range), mg/L	94 (1–407)
Chest X-ray changes, number of patients (% of total)	
*No CXR done (outpatients)*	17 (16%)
*No CXR changes:*	30 (28%)
*CXR changes consistent with COVID-19:*	49 (45%)
*Other/Indeterminate CXR changes:*	12 (11%)
Requirement for ICU (% of total)	
*Not required:*	69 (64%)
*Admitted to ICU:*	26 (24%)
*Assessed for ICU, deemed unsuitable:*	13 (12%)
Outcome	
*Full recovery*	82 (76%)
*Death*	10 (9%)
*Residual morbidity (including decreased independence)*	9 (8%)
*Outcome still unclear*	7 (6%)

**Table 2:** Disease characteristics.

Characteristic	
Total number of patients	108
Admitted to hospital, number of patients (percentage of total)	91 (84%)
Mild disease	17 (16%)
Moderate disease	51 (47%)
Severe disease	39 (36%)
Date of first SARS-CoV-2 positive sample, range	10/3/2020-2/5/2020
Interval between symptom onset and peak oxygen requirement, median (standard deviation), days	8 (6.3)
Requirement for supplemental oxygen, number of patients (percentage of total)	65 (60%)
Peak CRP, median (standard deviation), mg/L	94 (114)
Chest X-ray changes, number of patients (percentage of total)	
*No CXR done (outpatients)*	17 (16)
*No CXR changes:*	30 (28)
*CXR changes consistent with COVID-19:*	49 (45)
*Other/Indeterminate CXR changes:*	12 (11)
Requirement for ICU (percentage of total)	69 (64)26 (24)
*Not required:*	13 (12)
*Admitted to ICU:*	
*Assessed for ICU, deemed unsuitable:*	
Outcome (percentage of total)	
*Full recovery*	82 (76%)
*Death*	10 (9%)
*Residual morbidity (including decreased independence)*	9 (8%)
*Outcome still unclear*	7 (6%)

**Table 3:** Laboratory parameters.

Parameter	Reference Range	Cohort Results Median (IQR)
CRP mg/L	0–5	45.0 (7.4–101)
Haemoglobin g/dL	11.5–16.4	12.2 (10.4–13.7)
RDW	11–15	13.2 (12.5–15.2)
WBC x10^9^/L	4–11	5.6 (4.4–8.2)
Neutrophils x10^9^/L	2–7.5	3.5 (2.3–6.2)
Lymphocytes x10^9^/L	1.5–3.5	1.2 (0.8–1.7)
D-dimer ng/mL	0–500	831 (361–1616)
Fibrinogen g/L	1.9–3.5	4.4 (3.7–6.5)
Creatinine μmol/L	45–84	73 (61–92)
ALT IU/L	0–33	29 (17–59)
AST IU/L	0–32	32.5 (21–49)
Albumin g/L	35–50	35 (31–41)
Ferritin μg/L	23–393	451 (194–1023)
LDH IU/L	135–250	228 (190–303)
Triglycerides mmol/L	0.5–1.7	1.32 (1–1.78)
Interleukin 6 pg/mL	0.09–7.26	19.5 (6.5–40.5)
Soluble CD25 pg/mL	101.8–2509.4	1749.7 (1371–3289)
T cell count (CD3^+^) x10^6^/l	797–2996	825 (563–1231)
% T cells (CD3^+^)	66–85	69 (62–76)
Helper T cell count (CD3^+^CD4^+^) x10^6^/l	502–1749	505 (332–810)
% Helper T cells (CD3^+^CD4^+^)	35–60	45 (37–54)
Cytotoxic T cell count (CD3^+^CD8^+^) x10^6^/l	263–1137	264 (180–396)
% Cytotoxic T cells (CD3^+^CD8^+^)	18–49	21 (16–29)
B cell count (CD19^+^) x10^6^/l	99–618	161 (81–218)
% B cells (CD19^+^)	5–19	13 (7–18)
NK cell count (CD16^+^CD56^+^) x10^6^/l	72–577	161 (94–275)
% NK cells (CD16^+^CD56^+^)	4–24	12 (8–22)

### 2.2 Severe SARS-CoV-2 infection is associated with evidence of profound immune dysregulation

**Figure 1:**
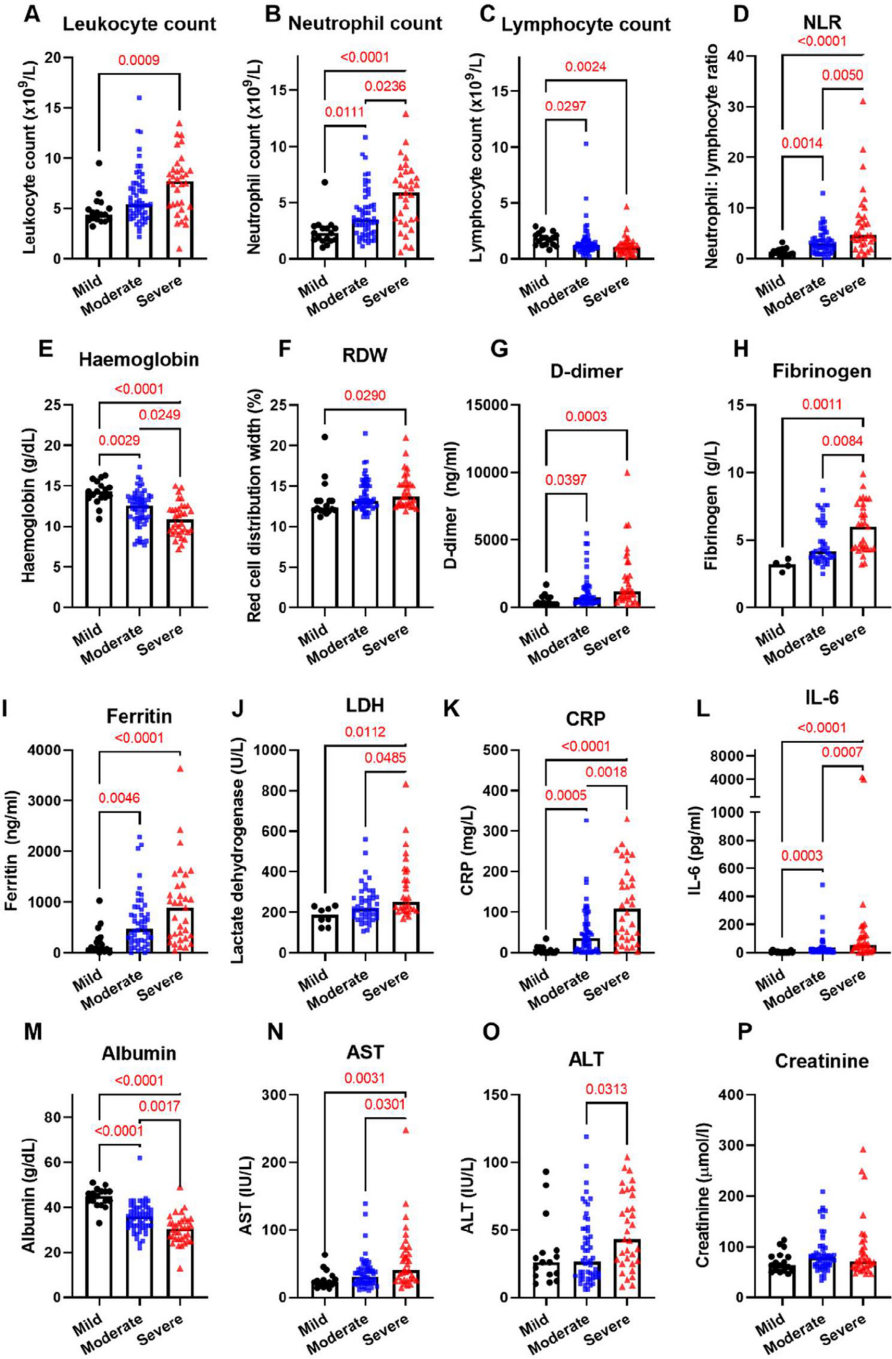
**Severity of acute COVID-19 and Markers of Inflammation, Cell Turnover and Coagulation.** Severe COVID-19 is accompanied by **(A)** leukocytosis **(B)** lymphopenia **(C)** neutrophil and **(D)** increased neutrophil: lymphocyte ratio in disease (N = 108 total). Severe COVID-19 is also associated with **(E)** lower haemoglobin **(F)** greater red cell distribution width **(G)** increased D-dimer **(H)** increased fibrinogen. Severe COVID-19 was associated with increasing **(I)** LDH **(J)** Ferritin **(K)** CRP **(L)** IL-6, **(M)** lower albumin **(N)** increased AST **(O)** increased ALT. No change in **(P)** creatinine with severity.

Analysis of routine laboratory tests revealed widespread changes in leukocyte populations with a marked coagulopathy and increased markers of cell turnover and inflammation (Figure 1). The degree of lymphopenia significantly increased with disease severity, while marked neutrophilia was seen particularly in those with severe disease. Anaemia with a left shift, characterised by increased red cell distribution width, was also noted with severe disease. Greater disease severity was associated with greater D-dimer and fibrinogen levels, as well as markers of cellular inflammation (CRP, IL-6, ferritin) and cell turnover (LDH).

**Figure 2:**
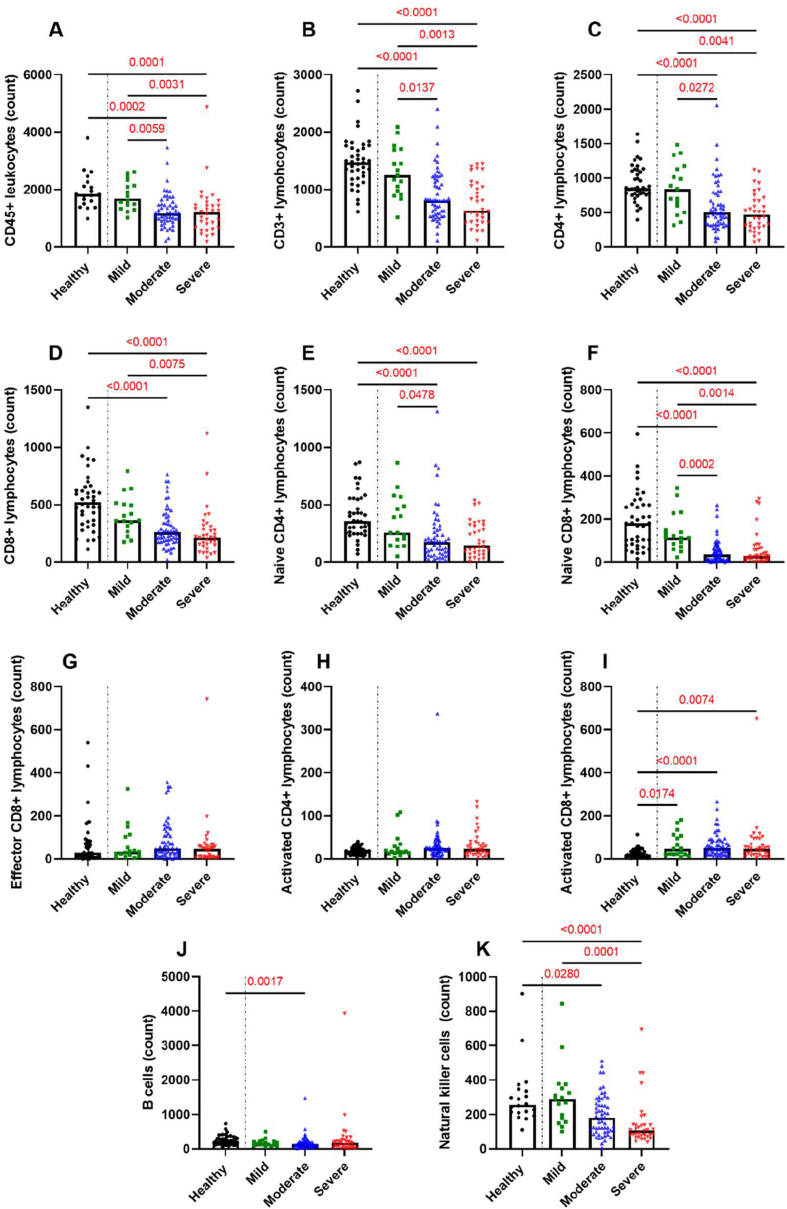
**Analysis of Major Lymphoid Subsets Reveals a Widespread Lymphopenia in COVID-19 Disease.** Peripheral blood immunophenotyping in those with mild, moderate and severe coronavirus disease revealed significant decreases in **(A)** CD45 positive cells (leukocytes), most pronounced in moderate/severe disease; significant decreases in **(B)** CD3, **(C)** CD4 and **(D)** CD8 cell counts, greatest in those with moderate/severe disease in comparison to controls; **(E)** naïve CD4 and **(F)** CD8 cells both significantly decreased in COVID-19 with increasing disease severity; whilst **(G)** effector CD8 cells were non-significantly elevated, there was a significant expansion in **(H)** activated CD4+ and **(I)** CD8+ T cells, which did not reflect disease severity. Both **(J)** B cells and **(K)** Natural Killer cells were significantly decreased in number in COVID-19. Decreases in Natural Killer cell number reflected disease severity.

In order to further delineate the panlymphopenia seen on the full blood count, detailed immunophenotyping of lymphoid cells was conducted (Figure 2). This confirmed panlymphopenia, with reduction in total CD3+ cells across all disease severities, most marked in those with severe illness. There was reduction in both CD4+ and CD8+ T cell counts in a similar pattern. On further subset analysis, there was global reduction of naïve CD4+ and CD8+ cells and increased activated CD4+ and CD8+ cells, as well as increased effector CD8+ cells across all disease states. The effect of disease severity was less marked in these subsets, with similar numbers of activated and effector cells across all patients, and similar naïve cell counts in both moderate and severe disease.

**Figure 3:**
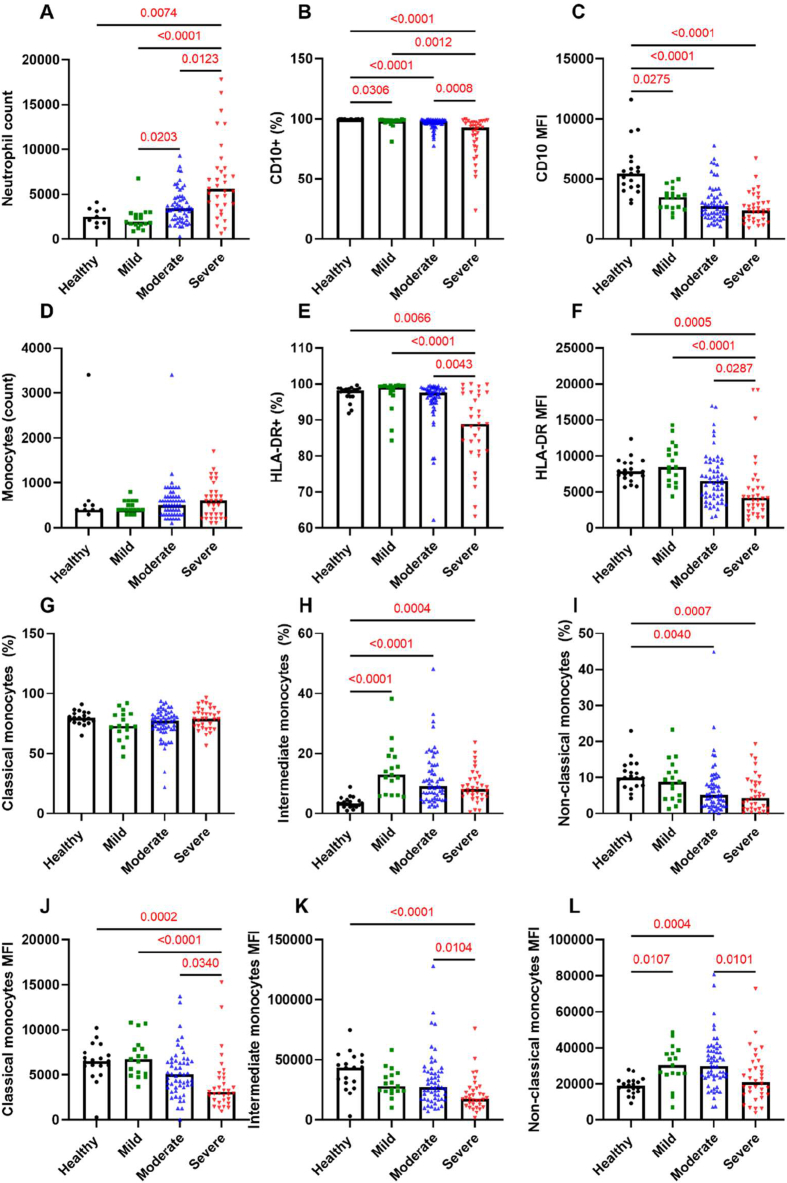
**Analysis of Myeloid Cells Reveals a Widespread Immune Dysregulation in COVID-19 Disease**. Peripheral blood immunophenotyping in those with mild, moderate and severe coronavirus disease revealed significant changes in neutrophil markers with **(A)** neutrophilia, **(B, E)** reduced neutrophil CD10 expression and **(C)** reduced neutrophil CD16 expression as well **(D, F)** reduced Mean Fluorescence Index (MFI) of these markers, most pronounced in those with severe COVID-19 disease. **(G)** Monocyte numbers were not significantly altered, but there were significant changes in monocyte subsets, with **(H)** reduced HLA-DR + monocytes, **(J)** no change in classical monocytes.lower number of non-classical monocytes, **(K)** increased intermediate monocytes **(L)** no change in classical monocytes. The MFI changes are shown in **(M-O)**. Monocyte subpopulations are shown as proportions of total monocyte population, while neutrophil CD10+ and CD16 + populations are shown as a proportion of total neutrophil population, based on flow cytometry gating.

Detailed immunophenotyping of myeloid populations was also carried out, given the profound neutrophilia noted on initial investigations (Figure 3). Marked severity-associated neutrophilia was confirmed. There was loss of CD10 and CD16 expression by neutrophils with increasing disease severity. This finding was confirmed by looking at both CD10 and CD16 expression on neutrophils as well as the median frequency intensity (MFI) of these markers. In addition to these neutrophil changes, perturbations amongst monocyte populations were found. While changes in the absolute number of monocytes were not associated with infection, there was progressive loss of HLA-DR expression by monocytes with increasing disease severity. This was again confirmed by two methods, using percentage HLA-DR expression and MFI. There was an increase in proportion of intermediate monocytes in SARS-CoV-2 patients when compared to healthy controls. This increase was most marked in those with mild disease. While there were no significant differences in non-classical monocyte proportions between infected and healthy patients, there were differences across disease severities, with milder patients having a higher proportion when compared to moderate and severe patients. The classical monocyte proportions remained unchanged when compared to healthy controls and when assessed across disease severities.

**Figure 4:**
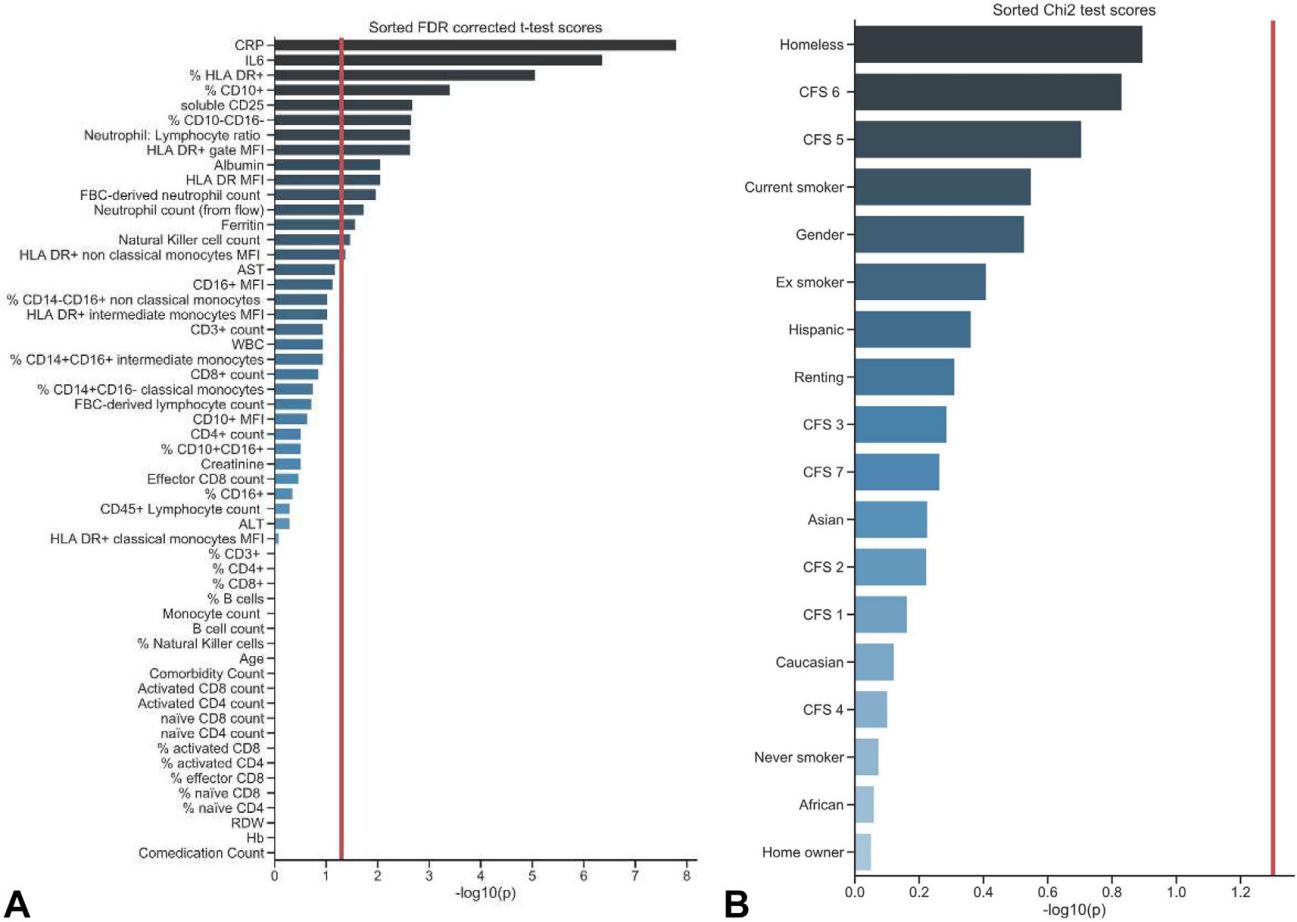
**Univariate analysis of numerical demographic, clinical and immunological variables associated with severe COVID-19. (A)**. t-test scores for differences in means between patients with severe and non-severe COVID-19 are indicated in the bar graph, corrected for false discovery rate using Benjamini–Yekutieli procedure The scores were derived from p-values by calculating -log10 (p-value). The red line indicates threshold of statistically significance (p < 0.05). Univariate analysis of categorical variables associated with severe COVID-19 **(B)** Sorted Chi-squared test scores were used to test for significant associations between categorical variables and severe COVID-19. The red line indicates threshold of statistically significance (p < 0.05).

Between-group differences in those with severe COVID-19 in and those with mild or moderate disease were assessed. Univariate analysis of the 71 variables in our clinical and laboratory dataset identified a series of 15 factors associated with severe COVID-19 that remained statistically significant when corrected for false discovery rate (Figure 4A). These are ranked by strength of association, with strong associations seen with some of the differences noted in our earlier analysis. Specifically, elevation of the acute phase reactants CRP and IL-6 were closely associated with severe disease, with increased soluble CD25 also seen with severe disease. Furthermore, reduced expression of the neutrophil marker CD10 and monocyte marker HLA-DR were strongly associated with severe disease. No lymphocyte marker approached significance. Chi-squared testing of categorical variables ranked homelessness, male gender, higher clinical frailty scores and being a current smoker as the categorical variables most strongly associated with severe COVID-19, although no association met the threshold for statistical significance (Figure 4B).

### 2.3 Machine learning identifies and ranks markers of severity

**Figure 5:**
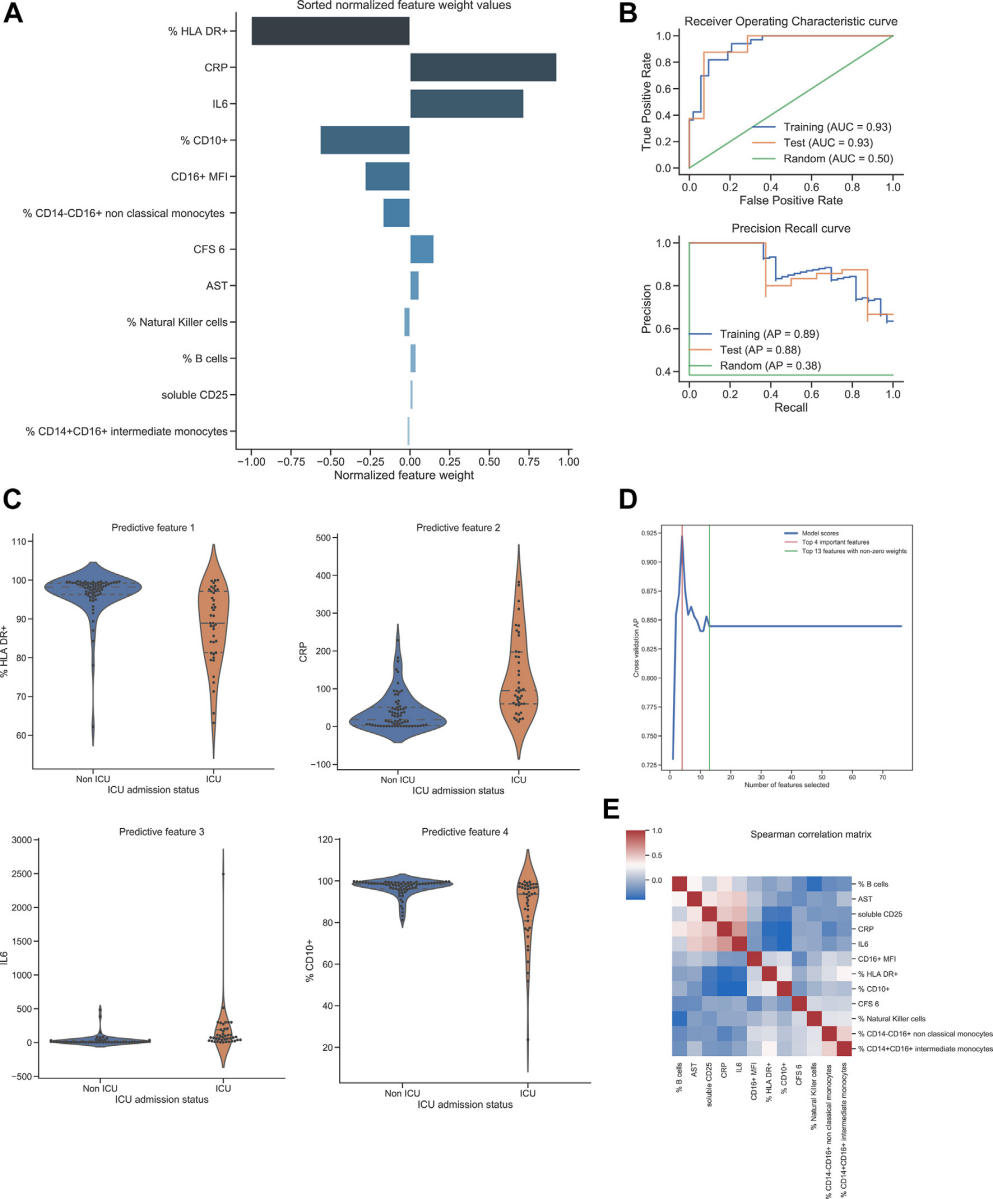
**Machine learning modelling of variables identifies immunological features as the strongest independent associations with severe COVID-19. (A)** Sorted normalised feature weight values of variables identified by the model as signatures of progression to severe COVID-19 (**B)** Model performance for training and test sets assessed using receiver operating characteristic (ROC) curves and precision-recall curves. **(C)** Violin plots representing the difference of values between severe and non-severe COVID for the four features with the highest weight values in the model **(D)** Diagnostic power of top four variables **(E)** Correlation matric of variables within the model, analysed using Spearman correlation. Blue indicates negative correlation between variables, red indicates positive correlation. Darker colour indicates a stronger association. Data was fitted to a logistic regression model with elastic net penalty. For the training set, the hyperparameters were optimized using a 10-fold cross-validation procedure and an exhaustive grid search on a training subset comprising 80% of the data. The remaining 20% of the data was used for the test set.

Having identified strong associations between specific individual immunological features and severity, multivariate analysis was used to establish the relative importance of the clinical and immunological parameters. A logistic regression model with elastic net penalty was applied to the data. The model demonstrated a high sensitivity and specificity for identification of severe disease. The area under the receiving operating characteristic (AUROC) curve was 0.93 on both training and test sets, indicating excellent model predictive performance and absence of overfitting (Figure 5B). These results were confirmed with a permutation test resulting in statistically significant output (Supplemental Figure 3A; p < 0.01). A precision-recall curve was also applied to estimate the model's predictive discriminating performance due to the class imbalance in the ICU outcome measure (39 versus 69 cases). The average precision (AP) score was 0.89 on the training set and 0.88 on the test set (Figure 5B). Both scores indicate excellent generalisation properties for this model. The model's true positive and true negative rates were assessed on training and test sets, visualized by confusion matrices (Supplemental Figure 3B), which indicate the model's excellent discrimination between classes and absence of overfitting.

When applied to the integrated dataset, the machine learning multivariate approach identified 12 variables associated with severe COVID-19 (i.e., with associated weights of non-zero values) when considered in interaction with all 71 variables. Normalised weight values associated with the top features of the model are shown in Figure 5A. The top four features stand out as most important with normalised weight values between 1.0 and 0.56 (Figure 5C). The normalised weight values of subsequent features were lower (0.28–0.01). The top feature indicative of severe COVID-19 was a reduced proportion of HLA-DR^+^ monocytes. The next most important features were higher CRP and IL-6 levels, and a lower percentage of CD10^+^ neutrophils (Figure 5A). Strikingly, a combination of these four features alone could identify severe COVID-19 with an average precision score of 0.925. Additional immunological features associated with severe COVID-19 with lower weight values included lower CD16 expression by neutrophils, lower percentages of non-classical (CD14^−^CD16^+^) and intermediate (CD14^+^CD16^+^) monocytes and lower numbers of NK cells (Figure 5A and Supplemental Figure 4A, 4B, 4H). Other parameters associated with severe COVID^-^19 were a high clinical frailty score of 6, higher AST levels, higher B cell counts and higher sCD25 levels (Figure 5A and Supplemental Figures 4C–G). Importantly, introducing additional variables to the four highest-ranked features did not improve the ability of the model to recognise severe COVID-19 (Figure 5D).

Potential relationships between each variable identified in the machine learning analysis were also investigated using a clustered Spearman correlation matrix (Figure 5E). Unsurprisingly, there was a positive correlation between levels of CRP and IL-6, with both markers negatively correlated with levels of HLA-DR^+^ monocytes and CD10^+^ neutrophils. Levels of HLA-DR^+^ monocytes and CD10^+^ neutrophils correlated positively with each other. There was a positive correlation between IL-6 and CRP with AST and sCD25. Proportional changes in non-classical and intermediate monocytes were also strongly associated with each other.

## 3 Discussion

COVID-19 has presented an enormous challenge to human health and society. Pre-morbid risk factors for the development of severe COVID-19 include older age, male gender, obesity, and co-morbidities cardiovascular disease, respiratory disease, diabetes, cancer and immunodeficiency are well recognised [32]. However, even amongst patients with several concurrent risk factors, severe disease is not ubiquitous. This heterogeneity in clinical outcomes of SARS-CoV-2 infection is likely to be driven by variation in immune responses [12]. There is emerging evidence, for instance, that host immune defects, including blunted antiviral type 1 interferon responses, are associated with poor outcomes [33, 34]. Identification and characterisation of the immune dysregulation in the subset of patients that develop severe disease is considered increasingly important. The nature of the early stages of immune dysregulation in COVID-19, and how this relates to other risk factors to affect disease severity, is an area that requires further investigation.

The data presented here on 108 patients with SARS-CoV-2 infection confirms experimental and research laboratory work on the characteristics of severe infection [35, 36, 37]. It includes patients across the spectrum of disease severities, from mildly symptomatic to those requiring mechanical ventilation; 69 (60%) patients required supplemental oxygen therapy, 39 (36%) progressed to severe COVID-19, and 17 (16%) had mild disease managed in ambulatory care. This disease spectrum is vital in drawing strong conclusions in terms of the role of severity indices.

Our cohort is broadly reflective of those reported elsewhere, with a median age of 61, more males than females requiring admission and a mortality rate of 9%, which was higher for men [38, 39, 40]. The similarities to cohorts reported elsewhere are important, given that early attempts to describe the immunopathological signatures of COVID-19 have been poorly applicable across populations [41, 42, 43].

Another strength of this study is the use of a whole blood flow protocol, with minimal handling, no PBMC isolation step and analysis within 4 h of blood draw. This minimized artefactual alterations in myeloid populations. Patients presented to hospital and had their bloods drawn a median of 2 days prior to peak COVID-19 as defined by peak oxygen requirement, giving us insight into the immune mechanisms leading to severe respiratory distress. In addition, all of our laboratory parameters were generated within a diagnostic laboratory. This means that results were generated in a clinically-relevant timeframe.

Severe disease in this cohort is characterised by elevated inflammatory markers and a shift towards emergency myelopoiesis. Those with severe illness demonstrate increases in the inflammatory cytokine IL-6, CRP and soluble CD25. This hypercytokinaemia was reported in some of the very early work from China, and confirmed with later studies [8, [44], [45]]. A strong association with immature myeloid cells, both neutrophils and monocytes, was seen with severe disease. This shift in myelopoiesis has been highlighted previously [35], with left-shift of myeloid cells along with increased cytokine production has been seen in early infection [46]. The emergence of anaemia and increased RDW in severe disease is observed, suggestive of emergency haematopoiesis. Indeed, increased RDW over the course of inpatient stay has been associated with increased COVID-19 mortality [47]. A combination of these features have been reported across several studies [48].

The machine learning model demonstrated evidence of inflammation and immune perturbation early in progression to severe COVID-19, with the emergence of a quartet of markers strongly associated with severe disease. These markers are seen before the onset of peak clinical illness in this cohort and were predictive of impending deterioration. The strongest single feature associated with severe disease on multivariate analysis was a reduced proportion of HLA-DR^+^ monocytes. Increasing data have emerged showing reduced monocyte HLA-DR expression is associated with critical illness in COVID-19 [21, 25, 53, 54]. Reductions in HLA-DR^+^ monocytes and reduced HLA-DR expression are appear to be an early predictor of poor outcome in other infectious states [49, 50, 51, 52]. Furthermore longitudinal studies indicate that ongoing decline in monocyte HLA-DR expression is associated with poor outcomes in all cause sepsis [55]. The reduction of HLA-DR positive monocytes in severe disease may be indicative of immunoparesis during this stage of the disease in severely affected individuals.

The second member of the quartet identified by this study as being strongly associated with severe disease is a proportional reduction in CD10^+^ neutrophils, another marker of emergency myelopoiesis. CD10 is a cell membrane metalloprotein that serves as a marker of neutrophil maturity [56]. Reduced neutrophil CD10 expression is suggestive of an immature and proinflammatory population. Immature neutrophils, characterised by lower or absent CD10, are immunostimulatory by promoting T-cell proliferation, survival and IFNγ production and have been associated with poor prognosis in sepsis [57, 58]. Loss of HLA-DR expression in conjunction with reduced neutrophil CD10 expression has been reported in severe COVID-19 by Schulte-Schrepping *et al.* [23, 55] This left shift in neutrophil populations with severe disease has subsequently been identified in other centres [59, 60]. The emergence of immature myeloid populations in severe COVID-19 may be in response to monocyte pyroptosis following inflammasome activation by SARS-CoV-2 [61].

The final two members of the quartet characterising severe disease are the acute phase reactants IL-6 and CRP. Elevated levels of IL-6 (and downstream protein CRP) are well-described as signs of poor outcome in COVID-19 [62, 63, 64, 65]. Elegant studies have highlighted a potent pro-inflammatory cytokine response, with prominent IL-6 elaboration, accompanied by a blunted type I and III interferon response that seems unique to SARS-CoV-2 infection [32, 66]. IL-6 also appears to be an important driver of monocyte HLA-DR loss, placing this cytokine at the centre of the immunopathological signature of COVID-19 [21].

Surprisingly, only a single categorical clinical variable, higher clinical frailty scores, emerged on multivariate analyses as associated with severity. The clinical frailty score captures biological, rather than chronological age and is a predictor of mortality in a wide-range of conditions [67, 68]. Remarkably, a large number of other expected variables, including age and gender, failed to emerge as risk factors for severity in this cohort – however, this must be qualified by the fact that this cohort included relatively few individuals with mild disease and does not rule out a role for these factors as risk factors for hospitalisation, for example.

This is a single centre study in a largely white Irish cohort of predominantly hospitalised patients. Validation of these findings in other centres and with larger samples earlier in the course of disease is desirable.

Understanding the mechanisms causing some individuals with SARS-COV-2 infection to progress to severe disease is required to inform therapeutic design. Through robust analysis of extensive demographic, clinical and immunological parameters captured early in disease trajectory, these parameters are ranked and adjusted for high-dimensional interactions and report a signature of severity which is characterised by inflammation (elevated IL-6 and CRP) and emergency myelopoiesis (reduced neutrophil maturity and monocyte HLA-DR expression). Our machine learning model allowed us to effectively integrate temporally linked clinical and laboratory data collected prior to peak severity to generate risk factors predictive of deterioration [69]. Other researchers have used similar approaches in the context of COVID-19. Some investigators have focussed on clinical parameters to predict deterioration. Other groups have carefully parsed detailed immunophenotyping and transcriptomic datasets to risk stratify COVID 19 patients [70, 71]. We were able to focus on complex data that was available from an accredited diagnostic immunology laboratory. These laboratory data could be used to inform clinical decision making in real time scenarios.

This study builds on prior work describing severe COVID-19 infection, demonstrating that these features of emergency myelopoiesis develop prior to the point of peak illness and are of central importance in progression to severe COVID-19. Patients with mild disease who did not require hospitalisation are included; these patients represent an understudied cohort. Furthermore, these approaches can be delivered in an accredited clinical laboratory as routine tests. This is of particular importance given the need for early identification of individuals who may benefit from therapeutic interventions [29]. This work emphasises the central role of expanded immature neutrophil and monocyte populations in the setting of COVID-19 and supports the measurement of these populations as part of severity assessments. Furthermore, it demonstrates that these investigations can be performed rapidly in a diagnostic laboratory, providing results in a timeframe that can impact clinical decisions. The use of targeted measurement of this signature of severe disease can help streamline the investigation of patients admitted with COVID-19 infection and identify those with severe disease.

## Declarations

### Author contribution statement

Liam Townsend: Conceived and designed the experiments; Analyzed and interpreted the data; Wrote the paper.

Adam H Dyer, Sultan Imangaliyev, Arthur White and Rachel M McLoughlin: Analyzed and interpreted the data; Wrote the paper.

Aifric Naughton: Performed the experiments; Wrote the paper.

Jean Dunne, Nollaig M Bourke, Cliona O'Farrelly, Niall Conlon and Clíona Ní Cheallaigh: Conceived and designed the experiments; Analyzed and interpreted the data; Contributed reagents, materials, analysis tools or data; Wrote the paper.

Rachel Kiersey and Dean Holden: Performed the experiments; Wrote the paper.

Aoife Mooney, Deirdre Leavy, Katie Ridge. Jamie Sugrue: Performed the experiments.

Mubarak Aldoseri, Jo Hannah Kelliher, Martina Hennessy, Declan Byrne, Catriona Doyle, Ruth O'Riordan and Anne-Marie McLaughlin: Contributed reagents, materials, analysis tools or data.

Paul Browne: Conceived and designed the experiments.

Christopher L Bacon and Ciaran Bannan: Conceived and designed the experiments; Wrote the paper.

Ignacio Martin-Loeches: Contributed reagents, materials, analysis tools or data; Wrote the paper.

Colm Bergin: Conceived and designed the experiments; Contributed reagents, materials, analysis tools or data; Wrote the paper.

### Funding statement

L. Townsend was supported by the Irish Clinical Academic Training (ICAT) Programme, the Wellcome Trust and the Health Research Board (Grant Number 203930/B/16/Z), the Health Service Executive, National Doctors Training and Planning and the Health and Social Care, Research and Development Division, Northern Ireland.

R. McLoughlin was supported by the a Science Foundation Ireland (SFI) Investigator Award (15/IA/3041).

N. Conlon and C. Cheallaigh were supported by an SFI grant (Grant Code 20/SPP/3685).

### Data availability statement

Data will be made available on request.

### Declaration of interests statement

The authors declare no conflict of interest.

### Additional information

No additional information is available for this paper.

## Figures and Tables

**Table:** STAR Methods

REAGENT or RESOURCE	SOURCE	IDENTIFIER
**Antibodies (Fluorophore, clone)**		
CD45 (V500-C, 2D1)	BD Biosciences	Cat#655873
CD8 (V450, RPA-T8)	BD Biosciences	Cat#560347
CD3 (APC-H7, SK7)	BD Biosciences	Cat#641415
CD4 (PerCP/CY5.5, SK3)	BD Biosciences	Cat#332772
CD45RA (PE, -)	BD Biosciences	Cat#556627
CD27 (FITC, -)	BD Biosciences	Cat#555440
CD197 (Alexa Fluor 657, 150503)	BD Biosciences	Cat#560816
HLA DR (FITC, L243)	BD Biosciences	Cat#347400
CD38 (APC, HB-7)	BD Biosciences	Cat#345807
CD14 (APC, MФP9)	BD Biosciences	Cat#345787
CD16 (PE, B73.1)	BD Biosciences	Cat#332779
CD10 (APC, HI10a)	BD Biosciences	Cat#332777
**Commercial Assays**		
IL-6 ELISA	R&D Systems	Cat#D6050
sCD25 ELISA	R&D Systems	Cat#DR2A00
BD TruCount	BD Biosciences	Cat#340334
**Software and Algorithms**		
Python 3.7	Python	www.python.org
BD FACSDiva v8	BD BioSciences	
FLO Jo v10	BD	www.flojo.com

**Supplemental Table 1:** 71 parameters included in analysis

Category	Parameter
Demographics	Age Ethnicity (Caucasian, Asia, African, Hispanic) Smoking (current/ex/never) Comorbidity Count Gender Clinical Frailty Score (1–7) Housing Status (homeless/renting/homeowner) Comedication count
Serum Measures	Haemoglobin, red cell distribution width (RDW) White blood cell count, neutrophil count, lymphocyte count Creatinine ALT, AST Albumin Ferritin IL6, sCD25 Neutrophil: Lymphocyte ratio
Flow Measurements -Lymphoid Population -Myeloid Population	% naïve CD4^+^ % naïve CD8^+^ % effector CD8^+^ % activated CD4^+^ % activated CD8^+^ Naïve CD4^+^ count Naïve CD8^+^ count Effector CD8^+^ count Activated CD4^+^ count Activated CD8^+^ count % CD3^+^ % CD4^+^ % CD8^+^ % B cells % Natural Killer cells CD3^+^ count CD4^+^ count CD8^+^ count B cell count Natural Killer cell count CD45^+^ Lymphocyte count % CD10^+^CD16^+^ %CD10-CD16-% CD10^+^ CD10% MFI % CD16^+^ CD16^+^ MFI Neutrophil count % HLA DR^+^ HLA DR MFI % CD14-CD16^+^ non classical monocytes % CD14^+^CD16^+^ intermediate monocytes % CD14^+^CD16- classical monocytes HLA DR^+^ non classical monocytes MFI HLA DR^+^ intermediate monocytes MFI HLA DR^+^ classical monocytes MFI Monocyte count HLA DR^+^ gate MFI


**Supplemental Table 2:** Timing of samples

Time period	Median days (range)
Symptom onset to first blood draw	6 (1–15)
First blood draw to peak oxygen requirement (n 69)	2 (0–14)
Blood draw for immunophenotyping to peak oxygen requirement (n 69)	1 (13 pre to 33 post)

## Appendix

### Subject Details

This study was carried out in accordance with the Code of Ethics of the World Medical Association [73]. Ethical approval was granted by the Tallaght University Hospital/St. James's Hospital Joint Research Ethics Committee (reference REC 2020-03). Informed consent was obtained from all participants. Patients with a positive real-time PCR test for SARS-CoV-2 on nasopharyngeal aspirates (cobas© SARS-CoV-2 test, Roche Diagnostics, Rotkreuz, Switzerland) at our institution were recruited over a two-month period (Supplemental figure 1). 71 demographic, clinical and immunological measures were recorded for participants enrolled in this study (Supplemental table 1). These included social and medical history (smoking status, alcohol and drug use, housing status, co-morbidities, medication use and self-reported date of symptom onset), and clinical frailty scores (adjudicated by the admitting physician) [74]. Routine haematological and biochemical laboratory results at the earliest time point after the first positive SARS-CoV-2 test, radiologist-reported chest X-ray results, and clinical parameters including peak oxygen requirement and peak CRP value (within fourteen days of positive SARS-CoV-2 test), assessment for ICU admission, admission to ICU and outcome were extracted from the electronic patient record. Mild COVID-19 was defined as no requirement for hospital admission (WHO scale 1 and 2), moderate COVID-19 as requirement for hospital admission (WHO scale 3 and 4) and severe COVID-19 as requirement for high-level respiratory support (high flow oxygen, invasive ventilation (WHO scale 5 and 6) [75].

### Methods Details

Immunophenotyping was carried out on fresh whole EDTA treated blood and samples were analysed on a FACS Canto II Flow Cytometer (BD San Jose USA), using BD DIVA v8 and FLO Jo v10 software. BD FACSCantoTM clinical software was used for acquisition of BD MultiTestTM 6-colour TBNK and TruCount tubes. All other immunophenotyping samples were analysed using BD DIVA v8 and FLO Jo v10 software. T cell (CD4^+^ and CD8^+^), B cell (CD19^+^) and Natural Killer cell (CD16^+^CD56^+^) percentages and absolute counts were generated by standard methods (BD TruCount). Naive (CD27^+^) and Effector (CD27-) T cells were characterised using CD27, CD45RA and CD197 antibodies. T cell activation was assessed by CD38 and HLA-DR expression. These assays were previously validated and accredited in line with ISO15189 standards. Classical, intermediate and non-classical monocytes were characterised by CD14 and CD16 expression. Proportions of these subpopulations were reported as percentages of the total monocyte population. Neutrophil functional maturity was interrogated by evaluating CD10 and CD16 expression. Proportions of these populations were reported as percentages of the total neutrophil population. Cell phenotyping assays were validated and accredited in line with ISO15189 standards. Results were compared to a cohort of 40 pre-pandemic healthy controls (22/40 (55%) female; median age 48 (IQR 33–61), 20 of whom had extensive analysis performed for lymphoid and myeloid cell subsets, with 20 controls having extensive lymphoid analysis only. IL-6 and soluble CD25 (sCD25) levels were measured in serum by Quantikine™ ELISA kit (R&D Systems, Minneapolis, USA). 100μl of assay diluent and 100μl sample or control were added to each well. The assay was validated using 30 healthy volunteer serum samples to establish a normal range mean +3 SD. For validation, 25 samoles were also tested at another accredited site, Sheffield Immunology Laboratory, UK. Five NEQAS EQA controls were also included. All comparisons passed for accreditation.

### Statistical Analysis

Between group differences were examined in the first instance using standard univariate statistics (t-tests, Wilcoxon rank-sum and chi-square tests as appropriate based on normality testing). In order to compare differences across the three groups of increasing COVID-19 severity, Kruskal-Wallis testing with a Dunn's post-hoc test was used to estimate p-values. An alpha level of p < 0.05 was considered statistically significant.

A total of 71 demographic, clinical, laboratory and immunophenotyping parameters were analysed to assess associations with severe COVID-19 (Supplemental table 1). All data analysis, visualizations and modelling were performed using open source packages available in Python 3.7 [76, 77]. For univariate analysis, a FDR corrected t-test was applied on power transformed numerical variables. The power transformation was implemented using Yeo-Johnson transformation to ensure normal distribution of variables. FDR-correction was implemented using Benjamini-Yekuteli procedure [78]. Categorical variables were tested using Chi [2]-test. An elastic net regularized logistic regression model was used for multivariate analysis and implemented for simultaneous feature selection and ICU status prediction [79]. To train each model, the hyperparameters were optimized using a 10-fold cross-validation procedure and an exhaustive grid search on a training subset comprising 80% of the data. Generalization of the trained model was then assessed using the remaining 20% of the data as a test set.

Supplemental Figure 1: Patient enrolment flow chart

Supplemental Figure 2: Flow cytometry gating strategy. (A) Naïve and effector T lymphocytes **(B)** activated T lymphocytes **(C)** neutrophils **(D)** monocytes

Supplemental Figure 3: Performance of machine learning model. (A) Permutation test of model **(B)** Confusion matrices, showing performance of model on training and test sets

Supplemental Figure 4: Violin plots of predictive features 5–12. (A) CD16 MFI **(B)** Proportion of non-classical monocytes **(C)** Clinical Frailty Score **(D)** Aspartate transaminase **(E)** Proportion of natural killer cells **(F)** proportion of B cells **(G)** Soluble CD25 **(H)** Proportion of intermediate monocytes. MFI = median frequency intensity
