# Supplementary material for: Severe COVID-19 is characterised by inflammation and immature myeloid cells early in disease progression
Source: Heliyon. 2022 Apr 1;8(4):e09230. doi: 10.1016/j.heliyon.2022.e09230 (PMC8973020; doi:10.1016/j.heliyon.2022.e09230)

Supplemental figures

Supplemental Figure 1

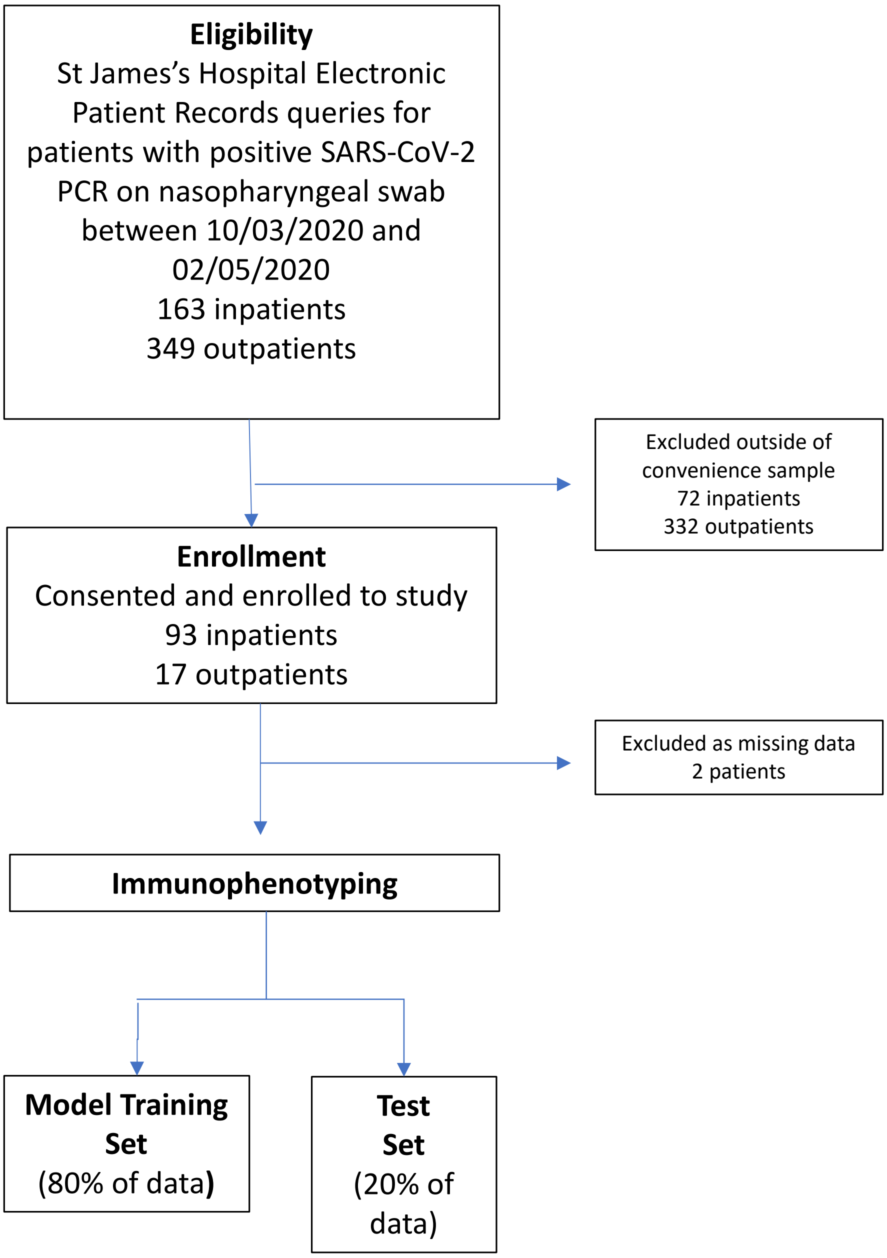

Supplemental Figure 2

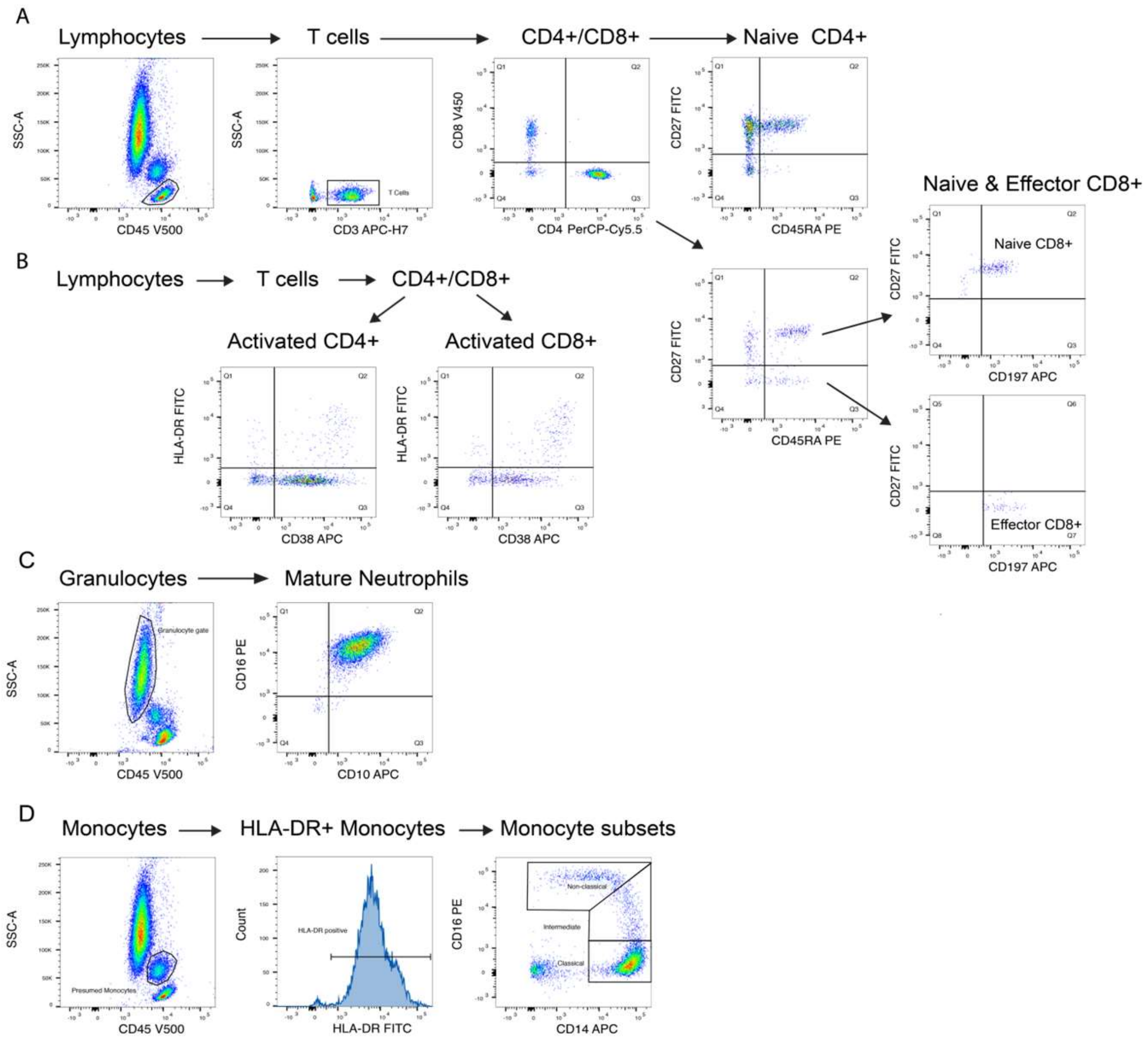

Supplemental Figure 3

(A)

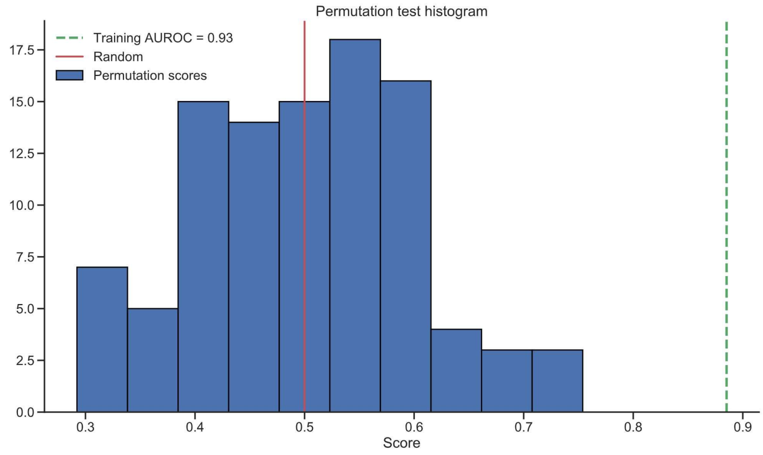

(B)

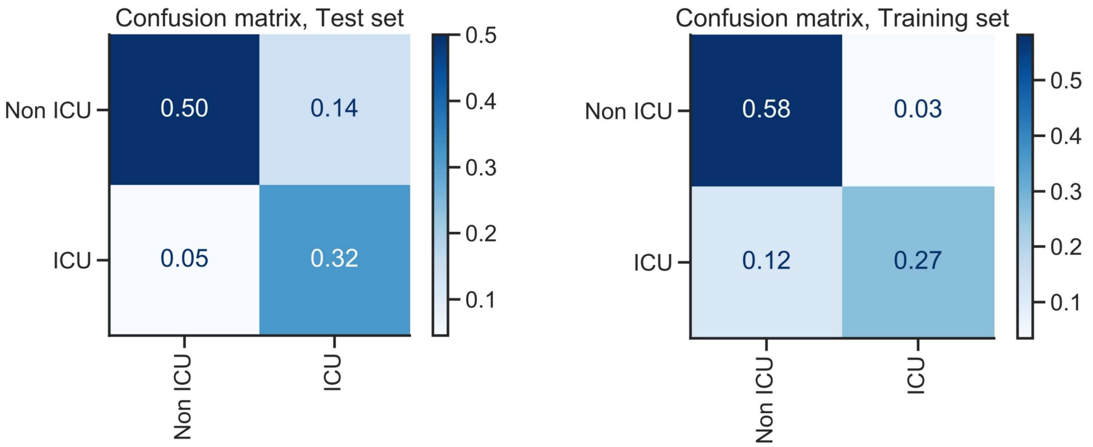

Supplemental Figure 4

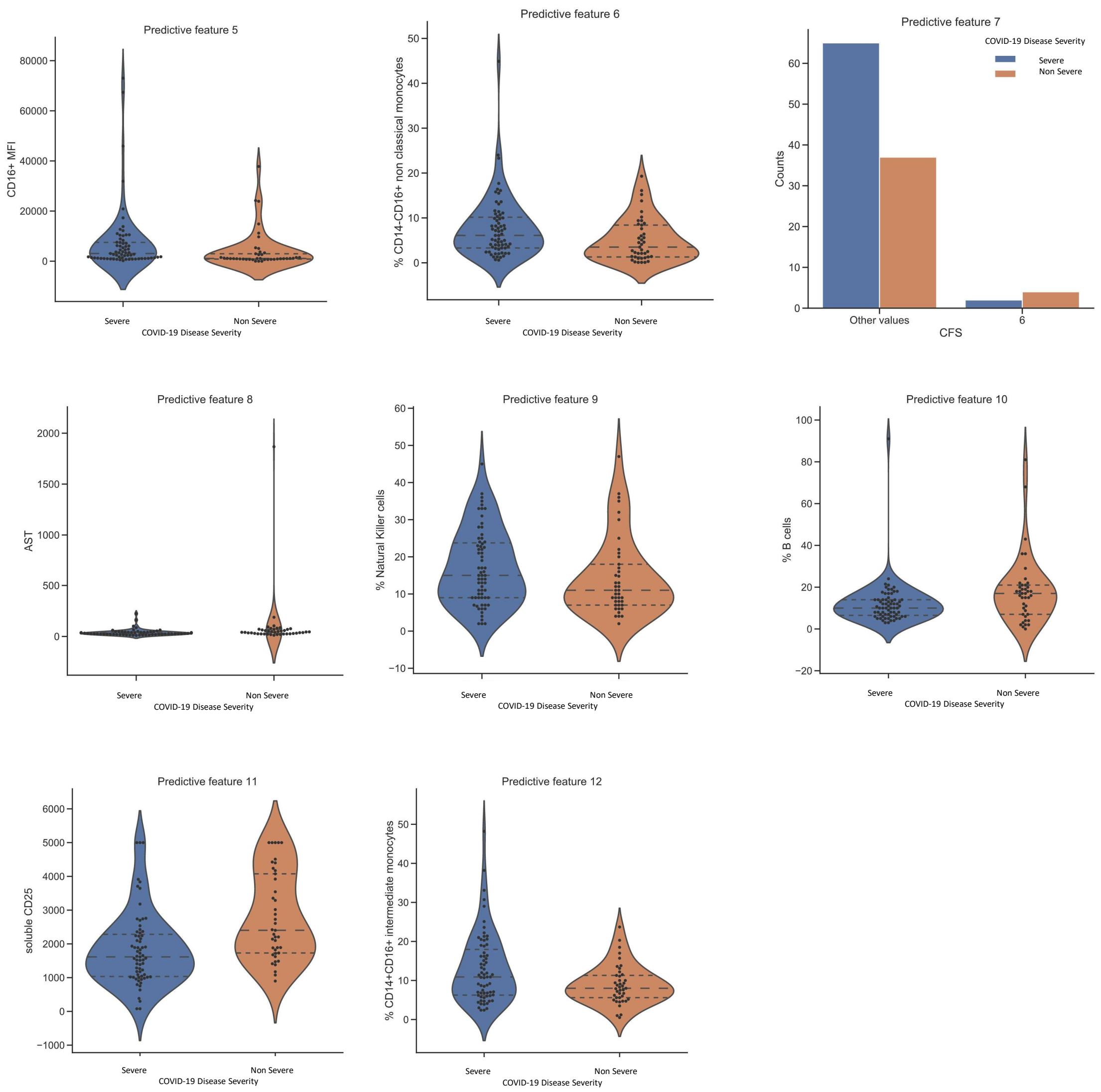

Supplement: Supplemental figures [file mmc1.pdf]
